# A Comparative Study of the Clinical Laboratory Quality Control Performance of AI-PBRTQC and Traditional PBRTQC Model in Tumor Marker Testing

**DOI:** 10.3390/diagnostics16101438

**Published:** 2026-05-08

**Authors:** Bowen Su, Yanpeng Zhang, Xian Wu, Yaping Jiang, Yinan Song, Xiaomin Shi

**Affiliations:** 1 Department of Laboratory Medicine, Peking University First Hospital, Beijing 100034, China; 2410108123@stu.pku.edu.cn (B.S.); 2310301105@stu.pku.edu.cn (Y.Z.); wuxian891020@163.com (X.W.); zjwljyp.love@163.com (Y.J.); athrunsyn@163.com (Y.S.); 2 School of Basic Medical Sciences, Peking University, Beijing 100191, China

**Keywords:** PBRTQC, bias, quality control, artificial intelligence, machine learning, deep learning, laboratory management

## Abstract

**Background:** The accuracy of tumor marker testing is critical for clinical decision-making. Patient-based real-time quality control (PBRTQC), as a complementary approach to traditional internal quality control (IQC), has been widely adopted in clinical laboratories. With the rapid advancement of automation and artificial intelligence (AI) in recent years, a large number of AI-based PBRTQC optimization algorithms have emerged. This study compared Patient-based real-time quality control integrating neural networks and joint probability analysis (NN-PBRTQC), Patient-Based Pre-Classified Real-Time Quality Control (PCRTQC), and traditional PBRTQC to identify the optimal method for quality control of tumor marker testing. **Methods:** The study utilized clinical tumor marker testing data from Peking University First Hospital. Six common tumor markers were selected, and constant error (CE) and proportional error (PE) were introduced as measures of analytical error. The False Alarm Rate (FAR) was used to reflect the specificity of the algorithms, while the Trimmed Average Number of Patient Results Affected Before Error Detection (tANPed) was used to reflect their sensitivity, in order to compare the clinical performance of the different models. **Results:** Under the same desired FAR (DFAR) of 0.1%, NN-PBRTQC reduced the tANPed for the six tumor markers by an average of 62% compared to the traditional PBRTQC while maintaining the same FAR, which demonstrated superior sensitivity of error detection. Meanwhile, although PCRTQC strictly controlled the FAR, its tANPed was 23% higher on average than that of the traditional PBRTQC, which indicated insufficient sensitivity of error detection. **Conclusions**: NN-PBRTQC demonstrated superior comprehensive quality control performance in the comparison of six common tumor markers. While ensuring that the FAR does not deviate from the DFAR, it significantly reduces tANPed, such that it could meet the specificity and sensitivity requirements of clinical testing. It is expected to enable more efficient and accurate detection of tumor marker errors.

## 1. Introduction

Tumor markers are critical biomolecules in cancer diagnosis and therapy, and they come in a wide variety, including proteins, enzymes, hormones, and circulating tumor DNA (ctDNA) [1,2,3]. They are produced either by the tumor cells themselves or as a result of the body’s physiological response to the tumor cells, and are typically released into the bloodstream. Therefore, their levels can be detected through serum testing [1]. The accuracy of tumor marker test results is closely linked to the effectiveness of clinical decisions such as early cancer screening, auxiliary diagnosis and prognosis assessment [1,2], which places stringent demands on quality control during the testing process. Currently, the reagents used for tumor marker testing in clinical laboratories are subject to challenges regarding the consistency and stability of test results due to the characteristics of calibration standards and inherent variations between batches [3].

Traditional internal quality control (IQC) relies heavily on quality control materials, which suffer from significant matrix effects, high long-term costs, and fixed monitoring cycles [4]. As a result, it struggles to meet clinical demands for real-time and continuous quality monitoring. Patient-Based Real-Time Quality Control (PBRTQC), leverages real-time monitoring based on patient test data, low cost and the absence of matrix interference, is gradually becoming a key supplementary method to address the shortcomings of traditional quality control [5,6,7]. Over years of development, PBRTQC has established a statistical algorithm framework that includes methods such as Moving Average (MA) and Exponentially Weighted Moving Average (EWMA) [8], and is now widely used in clinical laboratories. However, tumor marker test results exhibit a wide range of variation and a pronounced skewed distribution, which makes them prone to issues such as excessively high False Alarm Rate (FAR) or delayed error detection [3]. This severely hinders the widespread application of PBRTQC in tumor marker quality control.

In recent years, the rapid development of automation and artificial intelligence (AI) has driven the transformation of laboratory management toward intelligent and efficient operations [9]. The fields of academia and industry have deeply integrated AI, particularly machine learning (ML), using PBRTQC to develop numerous AI-PBRTQC optimization algorithms, such as Regression-Adjusted Real-Time Quality Control (RARTQC), Patient-Based Pre-Classified Real-Time Quality Control (PCRTQC), Patient-based real-time quality control integrating neural networks and joint probability analysis (NN-PBRTQC) and Patient-Based Graph-Based Anomaly Detection for Real-Time Quality Control (PGADQC) [10,11,12,13]. This provides diverse options for quality control in tumor marker testing. However, in practical applications, different AI-PBRTQC algorithms exhibit significant differences in sensitivity, specificity and applicable settings. More critically, most of these algorithms were developed independently by different research teams, and there is a lack of systematic comparative studies based on the same database. Furthermore, the absence of unified standards in existing comparative studies makes it difficult to intuitively present the strengths and weaknesses of each algorithm, so it is challenging for laboratories to make reasonable choices when faced with a variety of algorithms [14].

Currently, there is a relatively well-established consensus regarding the selection of PBRTQC algorithms, parameter optimization, and clinical applications in the field of biochemistry [15,16,17]. In previous studies, comparative analyses of different PBRTQC algorithm models have been conducted for clinical analytes such as electrolytes, blood cells, total cholesterol (TC) and low-density lipoprotein cholesterol (LDL-C) [14,18,19,20], which provides a robust foundation for the selection and application of PBRTQC. By contrast, research on PBRTQC for immunological assays, particularly in the field of tumor markers, remains largely unexplored [15,16,17]. There is an urgent demand to conduct targeted model comparative studies based on such research frameworks. Furthermore, compared to routine analytes such as electrolytes, tumor marker data exhibit more pronounced skewed distributions and a wider range of normal physiological variation. This fundamental difference imposes higher demands on models regarding their ability to detect systematic errors, resist interference, and control FAR. It also means that conclusions from model comparisons in routine laboratory tests cannot be directly applied to the field of tumor markers, making specialized model performance comparison studies for tumor markers an urgent priority.

The core innovation and value of this study lie in the fact that it is the first to utilize a large-scale dataset of tumor marker test results and employ a standardized evaluation system consistent with previous studies on conventional laboratory tests for comparative assessment. We selected two AI-optimized models, NN-PBRTQC and PCRTQC, which currently show high potential for clinical application, and compared them with the traditional PBRTQC. We systematically compared the performance differences among the three models in the detection of common tumor markers and conducted a multidimensional evaluation based on the clinical application requirements of tumor markers. The findings of this study could provide evidence-based guidance for clinical laboratories in developing and selecting precise, efficient tumor marker PBRTQC protocols, ultimately enhancing the quality, stability, and clinical utility of tumor marker testing.

## 2. Materials and Methods

### 2.1. Data Collection

**Figure 1:**
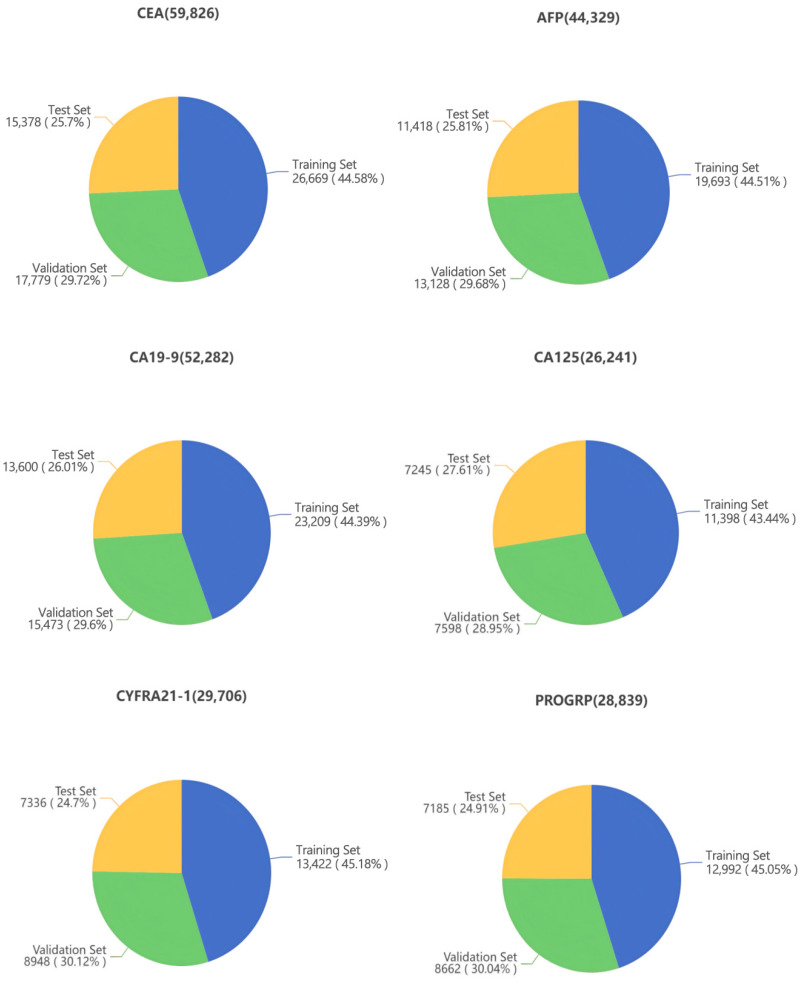
Distribution of datasets for six tumor markers (n).

The data were sourced from clinical tumor marker test records at Peking University First Hospital (2 January 2025 to 5 November 2025), with a focus on test results from the E801 instrument manufactured by Roche Diagnostics (Shanghai) Co., Ltd., Shanghai, China. The data covered six common tumor markers, including carcinoembryonic antigen (CEA), alpha-fetoprotein (AFP), carbohydrate antigen 19-9 (CA19-9), carbohydrate antigen 125 (CA125), cytokeratin 19 fragment (CYFRA21-1), and pro-gastrin-releasing peptide (PROGRP). Core information such as barcode numbers, patient category, gender, age, department, diagnosis, test results, test date and testing instrument was retained, while removing irrelevant and redundant information and filtering out abnormal records with missing, duplicate, or invalid data. All test specimens were serum samples, and all data underwent routine laboratory quality control to ensure the absence of significant human error or specimen handling errors. Additionally, all data were anonymized and approved for use to ensure the protection of participant privacy, in strict accordance with medical ethics standards. The dataset was arranged chronologically. Data from January to August were selected for the training and validation sets, with 60% allocated to the training set and 40% to the validation set, while data from September to November were used for the test set. The distribution is shown in Figure 1.

### 2.2. Basic Characteristics of the Data

The overall characteristics of the six tumor markers included in our study are shown in Table 1. The medians for all markers were significantly lower than the midpoint of their respective fluctuation ranges, and most data points were concentrated at the lower end of the range, indicating a clearly skewed distribution. Furthermore, there were significant differences in the distribution of test results among the different tumor markers. For example, PROGRP had the widest range (3.00–5000 pg/mL) and the highest degree of dispersion, while CYFRA21-1 had the narrowest range (0.1–500 ng/mL). Furthermore, CA19-9 and CYFRA21-1 exhibited the most pronounced skewness, which made them more difficult to detect.

### 2.3. Bias Introduction

In PBRTQC research, biases are often introduced to simulate instrument malfunctions for comparative performance testing of algorithms. In accordance with the Health Industry Standard WS/T403-2012 of the People’s Republic of China [21] and External Quality Assessment Programs in Laboratory Medicine from the National Center for Clinical Laboratories (NCCL) [22], we established a total allowable error (TEa) of 25% for each of the six tumor markers. For the test set, we introduced biases of 0, ±1, and ±2 times the TEa, including two types of biases: constant error (CE, Equation (1)) and proportional error (PE, Equation (2)):

(1) $$CE:x^{\prime }=x+n\times TEa\times Median(x)$$

(2) $$PE:x^{\prime }=x\times (1+n\times TEa)$$

±1 TEa represents the clinically acceptable error threshold, which can be used to evaluate an algorithm’s ability to detect minor biases from the threshold, given the high biological variability inherent in tumor markers [6,7]. Meanwhile, ±2 TEa represents a severe bias beyond the clinically acceptable range, simulating settings of severe laboratory malpractice to assess the algorithm’s performance in detecting major errors [6,7]. Due to the high variability and significant fluctuations of tumor markers, a ±0.5 TEa offset signal is easily masked by inherent random noise, making it impossible to effectively distinguish between systematic errors and normal physiological fluctuations. Consequently, it has no practical significance for quality control evaluation, and thus a ±0.5 TEa offset gradient was not included in this study. Furthermore, due to the pronounced skewness in the distribution of tumor markers, this experiment uses the median rather than the mean to calculate the CE.

For each of the six tumor markers, 10 independent test sets were sampled for each bias type, with each test set containing 3000 consecutive test data points starting from a random point. Subsequently, for each test set, the starting position of the bias was randomly generated. No bias was introduced before the starting point. After the starting point, the test values were adjusted according to the preset error type and magnitude. If the adjusted values exceeded the natural fluctuation range of the test, they were clipped to the upper and lower limits to facilitate the subsequent normal testing process. During the bias introduction process, the sampling starting position, error type, error magnitude, error starting position, and corresponding barcode numbers were recorded simultaneously to ensure data traceability.

### 2.4. Performance Evaluation Methods

Given the need to consider the specific clinical applications of PBRTQC, this study conducts a comparative analysis based on the two dimensions of specificity and sensitivity.

#### 2.4.1. Specificity

The FAR refers to the percentage of false alarms out of the total number of detections when there are no errors, and is used to evaluate an algorithm’s ability to avoid false alarms in the absence of true errors [8]. A lower FAR indicates higher specificity of the algorithm. The Desired False Positive Rate (DFAR) is a critical parameter for algorithm training and optimization. The optimal parameter must ensure that the FAR of both the training set and validation set does not exceed the DFAR, with performance verified using the test set [12]. In our study, the training and validation sets were trained with DFAR = 0.1%. Subsequently, for the test set with no TEa bias, the number of false alarms generated by the algorithm in the absence of true errors was recorded. We calculated the FAR for each of the 10 tests, summed, and averaged the results to obtain the actual FAR for the algorithm.

#### 2.4.2. Sensitivity

The Average Number of Patient Results Affected Before Error Detection (ANPed) is used to quantify the number of patient test results affected by an error during the period from when the error occurs until it is successfully detected [23]. A smaller ANPed indicates that the algorithm can detect errors more quickly and has higher sensitivity. tANPed (Trimmed Average Number of Patient Results Affected Before Error Detection) is a further refinement of ANPed that reduces the interference of outliers on the results by removing extrema from the Number of Patient Results Affected Before Error Detection (NPed) [8]. In our study, for test sets with ±1 and ±2 times the TEa, the NPed was calculated for 10 tests, and the model’s tANPed was obtained by summing and averaging the NPeds after excluding the maximum value. Furthermore, using tANPed as the core performance metric, we employed a paired *t*-test to conduct paired statistical comparisons of the detection performance of different models under various error settings, with a pre-set significance level of α = 0.05.

### 2.5. Model Principles

Our study selected two AI-PBRTQC models, PCRTQC and NN-PBRTQC, and compared them with the traditional PBRTQC. The principles of each model are described below.

#### 2.5.1. PBRTQC

Traditional PBRTQC directly uses the raw test results as input for statistical process control (SPC) and employs the EWMA algorithm for error detection. The weight λ in Equation (3) directly depends on N, λ=2/(N + 1), where N is the block size.

(3) $${EWMA}_{t}=\left(1-\lambda \right){EWMA}_{t-1}+\lambda x_{t}$$

Control limits are set at the upper and lower 0.05% percentile points to achieve a FAR of 0.1%. With a FAR of ≤0.1% and the minimum tANPed as the optimization objectives, all candidate parameter combinations are exhaustively searched. Only parameters that simultaneously satisfy the performance requirements of both the training set and the validation set are selected as the optimal combination, thereby avoiding overfitting in parameter selection and ensuring the stability and generalization ability of the model monitoring.

#### 2.5.2. PCRTQC

**Figure 2:**
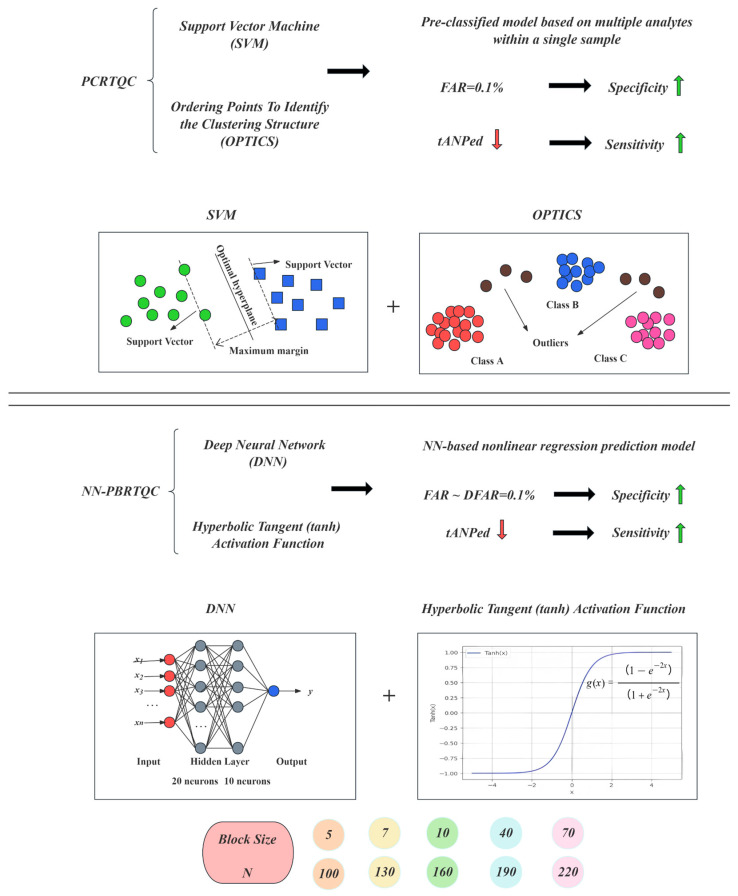
Model principles of PCRTQC and NN-PBRTQC. A red downward arrow indicates a decrease, while a green upward arrow indicates an increase.

As shown in Figure 2, the PCRTQC algorithm introduces a new step of pre-classification of patient samples prior to the traditional PBRTQC process. By leveraging clustering and classification tools such as Ordering Points To Identify the Clustering Structure (OPTICS) and Support Vector Machine (SVM) [24,25], it employs multi-analyte joint modeling to reduce intra-group data variability, thereby enhancing the performance of analytical error detection.

During the training phase, the companion analytes of the target analyte are first identified through association rule mining. Subsequently, samples containing both the target analyte and its companion analytes are selected, and data preprocessing is performed using multiple of median (MOM), logarithmic transformation, and inter-quartile range (IQR) normalization. The target analyte and its companion analytes are then mapped to a three-dimensional space, and stepwise clustering is performed using the OPTICS clustering algorithm. The resulting sample cluster labels are used as training data to construct an SVM classifier, with parameter tuning ensuring a classification accuracy of ≥90% [11]. The clustering results of our study are shown in Table 2.

During the testing phase, the SVM classifier is first employed to group samples. For samples without corresponding analytes, grouping is performed using maximum likelihood estimation combined with Bayes’ theorem. Subsequently, data in each group undergoes noise reduction via the Symlets series of wavelet transforms, normalization via the Box–Cox transformation, and z-score standardization, followed by mean filtering to further reduce data noise. Control limits are established based on the 0.05% and 99.95% percentiles of error-free data after filtering. Samples exceeding these control limits are classified as error samples, thereby enabling effective monitoring of errors [11].

#### 2.5.3. NN-PBRTQC

As shown in Figure 2, the NN-PBRTQC is an improved framework based on the traditional PBRTQC that integrates a deep neural network (DNN) with SPC [26,27]. Its core objective is to eliminate the interference caused by individual sample variations in error detection, thereby enabling efficient and precise monitoring of analytical errors.

The model first preprocesses the clinical testing data, removes outliers using the truncation method, and combines the Box–Cox transformation with z-score standardization to normalize the data and optimize comparability. Subsequently, a nonlinear regression neural network model incorporating patient demographic characteristics is constructed. The network architecture consists of one input layer, two hidden layers, and one output layer, with 20 and 10 neurons in the hidden layers respectively. All neurons utilize the hyperbolic tangent (tanh, Equation (4)) activation function.

(4) $$g(z)=\frac{1-e^{-2z}}{1+e^{-2z}}$$

Age, gender, clinic department, disease diagnosis, and baseline are used as input features. The neural network fits the nonlinear mapping relationship between these features and the test results, and calculates the residuals between the actual test results and the model’s predicted results. By substituting these residuals for the original test results as inputs to the SPC algorithm, we eliminate inherent sample variability caused by patients’ physiological and pathological characteristics, thereby enhancing the sensitivity of error detection [12].

Model training uses only the training set data, and employs the MATLAB R2019b Neural Network Toolbox to split the training set into a training subset, a validation subset, and a test subset in a 70%/15%/15% ratio. By applying a strict data partitioning strategy to constrain the training process, this effectively prevents the neural network model from overfitting. For error detection, the EWMA is used to monitor the residual time series, with control limits set at the upper and lower 0.05% percentile points, corresponding to a FAR of 0.1%. This study assumed historical monitoring data to be in a controlled state with no additional errors introduced, and the model was implemented using the MATLAB R2019b Neural Network Toolbox.

Parameter optimization is conducted with the dual objectives of keeping the FAR no higher than 0.1% and minimizing the tANPed. A brute-force search is performed on candidate parameter combinations for the block size (N = 5, 7, 10, 40, 70, 100, 130, 160, 190, 220), with simultaneous validation on both the training and validation sets. Only the optimal parameters that satisfy both criteria are selected to avoid overfitting due to parameter selection. Subsequently, the optimal parameter combination is applied to an independent test set to validate generalization performance, thereby further improving the specificity and sensitivity of the model’s error detection [12].

## 3. Results

### 3.1. Comparison of the Specificity of Different Algorithm Models

Under a DFAR of 0.1%, the FAR values were compared among NN-PBRTQC, PCRTQC and traditional PBRTQC, as shown in Table 3. The results indicated that for CEA, AFP, CA19-9, CA125 and CYFRA21-1, the FAR value for both NN-PBRTQC and traditional PBRTQC remained close to the DFAR, which met the model’s specificity requirements. However, for PROGRP, regardless of whether the introduced bias was CE or PE, both NN-PBRTQC and traditional PBRTQC were prone to FAR instability, with FAR value reaching 0.5%, which was significantly deviated from the DFAR. After adjusting the control threshold to 1.25 times the baseline, both methods reduced their FAR values to levels near the DFAR, which met the model’s specificity requirements. Subsequent tANPed statistics were calculated based on these adjusted values, involving one additional optimization step compared to other analytes to ensure the fairness of the comparison. Since all quality control methods to be compared must undergo head-to-head comparisons of error detection performance at equivalent FAR levels. By contrast, PCRTQC strictly controlled the PE and CE for all analytes to an FAR value of 0.1%, without any control failures, thereby ensuring the model’s specificity meets application requirements.

### 3.2. Comparison of the Sensitivity of Different Algorithm Models

**Figure 3:**
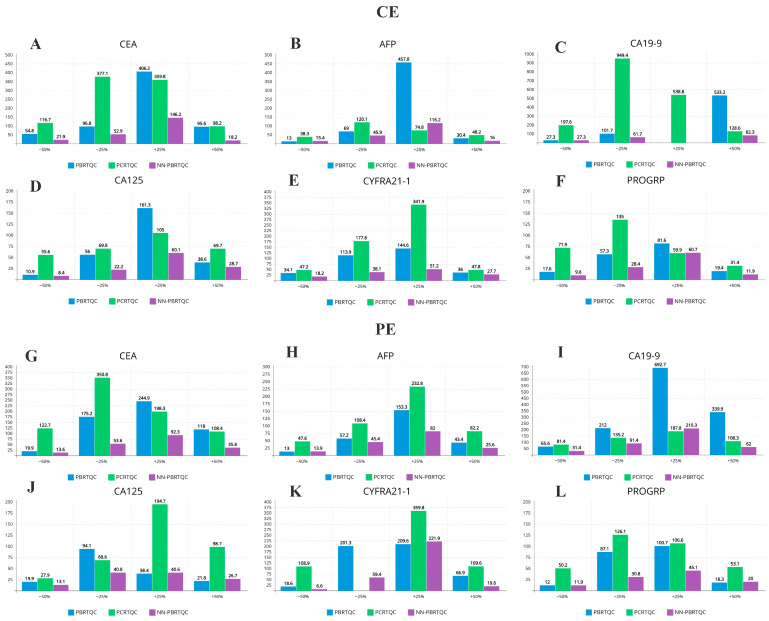
DFAR = 0.1%, comparison of tANPed values for CE (**A**–**F**) and PE (**G**–**L**) of each analyte by NN-PBRTQC, PCRTQC and PBRTQC. (PBRTQC and NN-PBRTQC failed to detect the CE of +1 TEa for CA19-9, while PCRTQC failed to detect the PE of −1 TEa for CYFRA21-1).

Under a DFAR of 0.1%, the tANPed values for NN-PBRTQC, PCRTQC and traditional PBRTQC were compared for TEa values of ±1 and ±2, as shown in Figure 3. Although PCRTQC demonstrated lower tANPed values than traditional PBRTQC under certain error conditions, and even slightly lower than NN-PBRTQC in optimal settings, its tANPed values were significantly higher in most other cases, with statistically significant differences (*p* < 0.05). Therefore, the analysis focused on comparing the performance of NN-PBRTQC and traditional PBRTQC.

**Figure 4:**
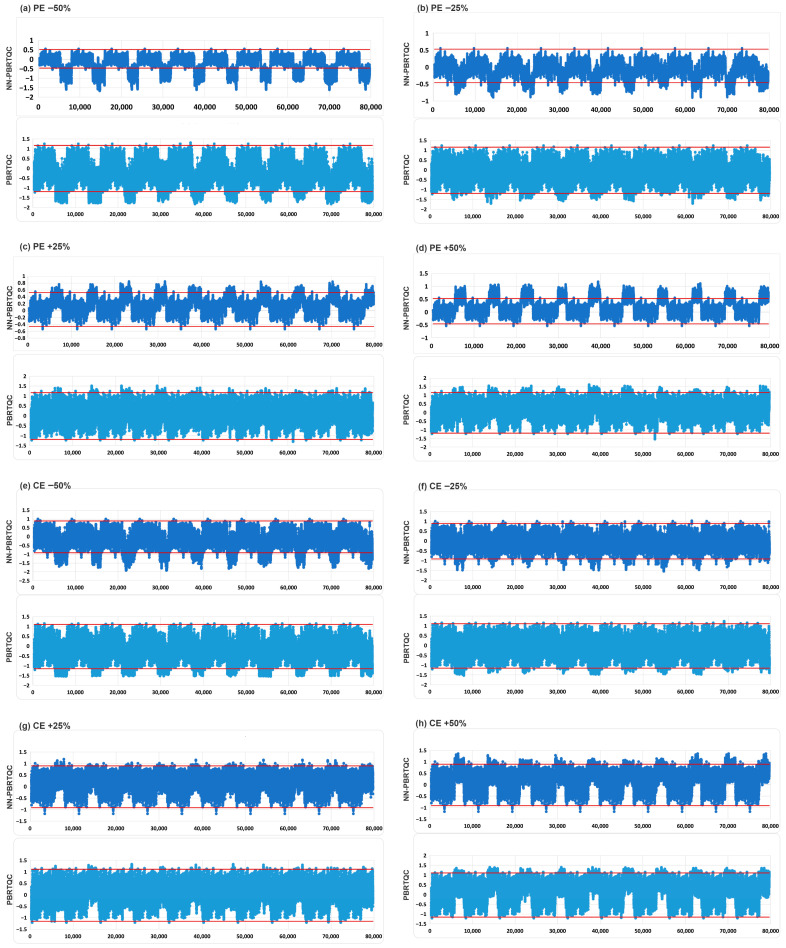
Comparison of quality control charts for PE (**a**–**d**) and CE (**e**–**h**) of CEA by NN-PBRTQC and PBRTQC. To simulate typical application scenarios, 5000 training data points were inserted before each set of 3000 data points in the 10 tests. The red line represents the control limit. If the PBRTQC value of a data point exceeds the red control limit line, an alarm is triggered. Therefore, the sensitivity of the model can be compared based on the ratio of the area of the portion of the quality control chart that exceeds the control limit to the total area. The area where NN-PBRTQC exceeded the control limits (red lines) was larger, indicating that NN-PBRTQC had better detection capability for CEA compared to PBRTQC.

Regardless of whether CE or PE was introduced, NN-PBRTQC demonstrated better sensitivity for CEA and PROGRP, with tANPed consistently lower than that of PBRTQC. Taking CEA as an example, for tANPed values of −2, −1, +1, and +2 TEa, the tANPed under CE was reduced by 60% (*p* = 0.024), 45% (*p* = 0.166), 64% (*p* = 0.035) and 81% (*p* = 0.054) respectively, while under PE, it was reduced by 32% (*p* = 0.196), 69% (*p* = 0.038), 62% (*p* = 0.055) and 70% (*p* = 0.030) respectively. The comparison of control charts for PE (a-d) and CE (e-h) of CEA by NN-PBRTQC and PBRTQC was shown in Figure 4. The area where NN-PBRTQC exceeded the control limits (red lines) was larger, indicating that NN-PBRTQC had better detection capability for CEA compared to PBRTQC.

**Figure 5:**
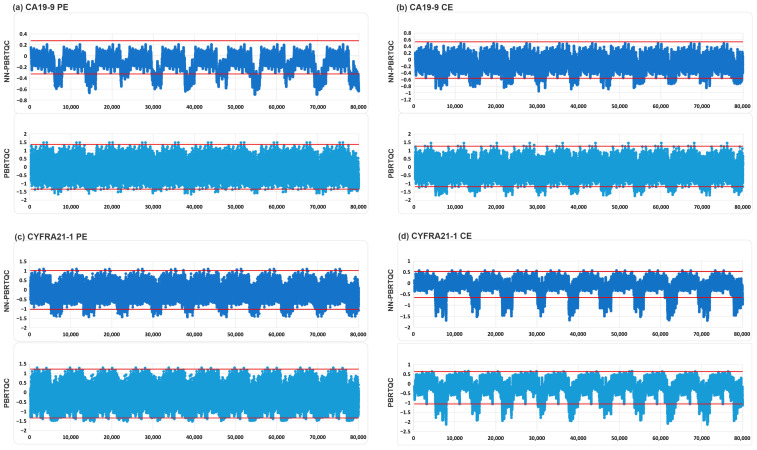
Comparison of PE and CE quality control charts for the −1 TEa of CA19-9 and CYFRA21-1 by NN-PBRTQC and PBRTQC. To simulate typical application scenarios, 5000 training data points were inserted before each set of 3000 data points in the 10 tests. The red line represents the control limit. If the PBRTQC value of a data point exceeds the red control limit line, an alarm is triggered. Therefore, the sensitivity of the model can be compared based on the ratio of the area of the portion of the quality control chart that exceeds the control limit to the total area. The area where NN-PBRTQC exceeded the control limits (red lines) was larger, indicating that NN-PBRTQC had better detection capability for the −1 TEa of CA19-9 and CYFRA21-1 compared to PBRTQC.

For AFP, CA19-9, CA125, and CYFRA21-1, NN-PBRTQC generally exhibited a significant overall trend of lower tANPed values compared to PBRTQC across most error settings, with the majority of these differences reaching statistical significance (*p* < 0.05). Only in a very small number of specific error settings were the tANPed values of NN-PBRTQC slightly higher than those of the traditional method. However, most of these numerical differences were not statistically significant (*p* > 0.05), and the number of such occurrences was far lower than the average daily sample volume in routine clinical practice, thus having no significant impact on the overall quality control evaluation. In particular, the optimization of −1 TEa error detection was more significant for CA19-9 and CYFRA21-1. For CA19-9, the tANPed corresponding to CE decreased from 101.7 to 52.9 (a 48% reduction), while that for PE decreased from 212 to 91.4 (a 57% reduction). For CYFRA21-1, the tANPed corresponding to CE decreased from 113.9 to 38.1 (a 67% reduction), while that for PE decreased from 201.3 to 59.4 (a 51% reduction). Although the differences between groups were not statistically significant (*p* > 0.05), there was still a noticeable trend toward clinical improvement. The comparison of PE and CE quality control charts for the −1 TEa of CA19-9 and CYFRA21-1 by NN-PBRTQC and PBRTQC was shown in Figure 5.

It was worth noting that for the CE of +1 TEa for CA19-9, both traditional PBRTQC and NN-PBRTQC showed not detected, while for the CE of +1 TEa for CYFRA21-1, PCRTQC also showed not detected. This demonstrated a limitation of the current technical system, which still struggled to detect minor biases in certain tumor markers.

## 4. Discussion

In recent years, with the ongoing integration of AI and PBRTQC, a large number of novel PBRTQC testing models have emerged [15,16,17]. This offers new insights into quality control in the field of laboratory testing, and a large number of AI-PBRTQC monitoring platforms have already emerged [20,28,29]. However, the strengths, weaknesses and application settings of these models vary significantly, and their detection capabilities for specific analytes require further comparative validation to assist clinicians in selecting appropriate models for clinical testing applications [14,30].

In 2023, Man et al. [11] constructed PCRTQC by performing sample pre-classification using OPTICS and SVM. This model does not require the truncation of outliers and can specifically reduce the FAR for specific populations. However, the threshold tuning for clustering algorithm is complex, requires samples to contain complete data for both target and companion detection analytes, and is susceptible to long-term systematic errors introduced by time series data. Additionally, models for special populations suffer from an abnormally high ANPed. In 2025, Xia et al. [12] proposed NN-PBRTQC, which leverages the feature extraction advantages of DNN in PBRTQC. This model effectively filters out interference from individual physiological variations, to ensure minimized ANPed under a predefined DFAR. However, the model relies on comprehensive, large-scale and multidimensional clinical data for training, and its detection performance declines significantly when data volume is small. Currently, these two models have been applied to quality control in certain specialized screening programs to optimize the specificity and sensitivity. However, there is a lack of relevant research regarding their application in tumor marker detection. Therefore, this study compares their performance with that of traditional PBRTQC to explore their potential for use in tumor marker detection.

Our results demonstrated that, for the tumor markers included in this study and under single-center experimental conditions, NN-PBRTQC exhibited superior overall performance in terms of error detection and quality control efficacy compared to PCRTQC and PBRTQC. For the six most common tumor markers, NN-PBRTQC had a slightly higher tANPed value than PBRTQC in only a very small number of error cases, and the difference was almost statistically insignificant (*p* > 0.05). In all other cases, NN-PBRTQC showed a clear trend toward reducing tANPed, with half of the cases exhibiting statistically significant differences (*p* < 0.05), which demonstrated its potential for improving quality control sensitivity. In particular, for minor errors of ±1 TEa, NN-PBRTQC was able to keep tANPed below 150 for CEA, AFP, CA125 and PROGRP, regardless of whether the error was a PE or CE, which was less than the daily sample volume. Even for CA19-9 and CYFRA21-1, which were inherently difficult to detect and exhibit significant fluctuations in detection sensitivity, NN-PBRTQC demonstrated a clear trend toward significantly shortening detection time, though no statistically significant differences were observed (0.05 < *p* < 0.2). By significantly reducing tANPed in the quality control process, sources of deviation can be identified and addressed more quickly in clinical applications, which greatly reduces the number of samples affected by errors and provides full assurance for the reliability of test results and precision medicine. Furthermore, aside from the uncontrolled FAR of PROGRP, which exhibited high variability and required manual adjustment to a control line of 1.25 times, the FAR of other tumor markers and the adjusted PROGRP can be strictly maintained near the DFAR, thereby meeting the specificity requirements of clinical testing. While PCRTQC can strictly control the FAR and resolve the issue of uncontrolled FAR for PROGRP, offering excellent specificity, in most cases, the tANPed was significantly too high, resulting in insufficient sensitivity. This severely limits its application in tumor marker testing.

The superior specificity and sensitivity demonstrated by NN-PBRTQC in experiments suggest potential applications for PBRTQC in the field of quality control for tumor marker testing. However, NN-PBRTQC still faces some practical challenges in real-world applications. For highly dispersed tumor markers such as PROGRP, FAR can easily spiral out of control. Under the initial model parameter conditions, the FAR in baseline testing reached 0.5%, requiring manual parameter tuning to return to normal levels. However, in actual clinical practice, it is impossible to predict in advance a latent increase in the FAR, and only after numerous abnormal alerts occur can the uncontrolled rise in the FAR be detected through retrospective, case-by-case investigation. This approach relies on manual intervention and experience-based parameter tuning, failing to achieve proactive adaptive steady-state control, which increases the consumption of human and material resources. Additionally, the model exhibited non-detection or high tANPed values for CA19-9 and CYFRA21-1, indicating that the model still has shortcomings in capturing minute errors in tests with high bias. Its sensitivity in identifying errors near the TEa threshold with small amplitude requires improvement. In the future, the NN-PBRTQC model can be further optimized to facilitate its broader application in tumor marker detection. Additionally, the field of deep integration between AI and PBRTQC has developed rapidly in recent years, with various emerging AI-PBRTQC algorithms continuously emerging. In future research, the latest AI-PBRTQC optimization algorithms can be included in comparative studies to identify algorithm models better suited for tumor marker detection.

This study employs the mainstream approach to algorithm comparison, which relies on introducing artificial errors to assess performance. However, this method can only predict the theoretical performance of the model and fails to reflect its actual capability in detecting real errors [15,31]. Therefore, we also deployed NN-PBRTQC and PBRTQC on a parallel computing platform established by the hospital’s laboratory department to validate their quality control applications. As a specific tumor marker commonly used in clinical screening for small cell lung cancer and neuroendocrine tumors, neuron-specific enolase (NSE) test results are highly sensitive to specimen pretreatment conditions and are easily affected by factors such as delays in blood centrifugation and specimen storage time. During routine testing, in response to a batch of NSE test results that showed elevated values due to delayed blood centrifugation, NN-PBRTQC issued an anomaly warning one day earlier than the traditional PBRTQC. This further validated the model’s superior sensitivity in real clinical scenarios, providing preliminary practical support for the simulation results of this study.

However, this study also has certain inherent limitations. Primarily, this is a single-center study, with all modeling, validation, and clinical data derived from a single medical institution. No independent external validation across institutions or testing platforms has been conducted. Further validation of the study’s generalizability is required using large-scale, multicenter data. Additionally, the study evaluated a limited range of tumor markers, and in real-world clinical applications, only NSE has generated alert instances to date, with coverage of other routine clinical tests yet to be expanded. Future efforts should expand the range of analytes included and conduct standardized external multicenter validation in collaboration with multiple medical institutions to gradually improve the model’s clinical applicability and value for widespread adoption. Furthermore, the overall statistical results indicate that only half of the differences between result groups reached a statistically significant level (*p* < 0.05). Although the remaining items showed a trend toward clinical optimization, they did not receive statistical support due to factors such as high variability in tumor markers, testing difficulties, and significant fluctuations. Future research could explore optimized evaluation methods that incorporate clinical decision-making weights to more accurately reflect the actual quality control value of different models.

## 5. Conclusions

Our study conducted a comparative assessment of the performance of AI-PBRTQC and traditional PBRTQC in the quality control of six common tumor markers. Overall, within the scope of analytes defined in this study and under single-center experimental conditions, NN-PBRTQC demonstrated superior performance to traditional PBRTQC in terms of sensitivity and specificity. It shows great potential for application in the crude quality control of tumor marker testing and can effectively improve the efficiency and accuracy of identifying testing errors. Given the objective limitations of this study, such as the single-center sample source and the limited number of analytes included, it is not yet appropriate to draw absolute conclusions regarding the model’s universal optimality. Further validation through subsequent multicenter studies and larger sample cohorts is required to confirm its clinical utility.

## Figures and Tables

**Table 1:** Overall data characteristics of the six tumor markers.

Analyte	Unit	Fluctuation Range	Mean		Median		SD		Skewness		Kurtosis	
			Training Set	Test Set	Training Set	Test Set	Training Set	Test Set	Training Set	Test Set	Training Set	Test Set
CEA	ng/mL	0.30–1000	6.05	5.57	1.92	1.81	46.06	40.60	21.39	19.20	652.01	421.62
AFP	ng/mL	0.91–1210	9.34	8.75	2.85	2.85	80.10	73.73	18.15	15.24	490.28	237.74
CA19-9	U/mL	2.00–1000	27.48	27.15	11.50	10.80	107.84	122.64	32.52	34.95	2699.22	2343.66
CA125	U/mL	1.00–5000	24.53	21.18	11.30	11.80	129.85	90.69	27.40	32.24	917.40	1459.62
CYFRA21-1	ng/mL	0.10–500	2.95	3.19	2.17	2.15	6.92	13.05	37.46	32.36	2072.48	1172.57
PROGRP	pg/mL	3.00–5000	66.66	65.52	47.60	46.20	203.33	202.08	20.26	20.54	450.96	469.43

**Table 2:** Companion analytes for each target analyte.

Target Analytes	Companion Analytes
CEA	AFP, CA19-9
AFP	CEA, CA19-9
CA19-9	AFP, CEA
CA125	CEA, CA19-9
CYFRA21-1	PROGRP, CEA
PROGRP	CYFRA21-1, CEA

**Table 3:** DFAR = 0.1%, comparison of FAR values for each analyte by NN-PBRTQC, PCRTQC and PBRTQC.

Analyte	Error Type	FAR		
		PBRTQC	PCRTQC	NN-PBRTQC
CEA	CE	0.19%	0.10%	0.10%
	PE	0.17%	0.10%	0.05%
AFP	CE	0.09%	0.10%	0.15%
	PE	0.06%	0.10%	0
CA19-9	CE	0.10%	0.10%	0.09%
	PE	0.08%	0.10%	0.06%
CA125	CE	0	0.10%	0
	PE	0.15%	0.10%	0.07%
CYFRA21-1	CE	0.11%	0.10%	0.11%
	PE	0.07%	0.10%	0.11%
PROGRP	CE	0.50% → 0.11%	0.10%	0.50% → 0.11%
	PE	0.50% → 0.07%	0.10%	0.50% → 0.11%

## Data Availability

The original contributions presented in this study are included in the article/Supplementary Material. Further inquiries can be directed to the corresponding author.

## Supplementary Materials

The following supporting information can be downloaded at: https://www.mdpi.com/article/10.3390/diagnostics16101438/s1, Table S1: Overall data characteristics of the six tumor markers; Table S2: Optimal parameter configurations for NN-PBRTQC and PBRTQC for each analyte; Table S3: Comparison of FAR ANPed, and tANPed values for each analyte by NN-PBRTQC, PCRTQC and PBRTQC; Table S4: Statistical analysis and comparison of tANPed values for various analytes by NN-PBRTQC, PCRTQC and PBRTQC.

## Abbreviations

PBRTQC	Patient-Based Real-Time Quality Control
IQC	Internal quality control
AI	Artificial Intelligence
NN-PBRTQC	Patient-based real-time quality control integrating neural networks and joint probability analysis
PCRTQC	Patient-Based Pre-Classified Real-Time Quality Control
CE	Constant error
RE	Relative error
FAR	False Alarm Rate
tANPed	Trimmed Average Number of Patient Results Affected Before Error Detection
DFAR	Desired False Alarm Rate
ctDNA	circulating tumor DNA
MA	Moving Average
EWMA	Exponentially Weighted Moving Average
ML	Machine learning
PGADQC	Patient-Based Graph-Based Anomaly Detection for Real-Time Quality Control
CEA	carcinoembryonic antigen
AFP	alpha-fetoprotein
CA19-9	carbohydrate antigen 19-9
CA125	carbohydrate antigen 125
CYFRA21-1	cytokeratin 19 fragment
PROGRP	pro-gastrin-releasing peptide
OPTICS	Ordering Points To identify the Clustering Structure
SVM	Support Vector Machine
MOM	multiple of median
IQR	inter-quartile range
DNN	deep neural network
SPC	Statistical Process Control
NCCL	National Center for Clinical Laboratories
TEa	Total Error allowable
ANPed	Average Number of Patients until Error Detection
NPed	Number of Patients until Error Detection
TC	Total cholesterol
LDL-C	Low-density lipoprotein cholesterol
NSE	neuron-specific enolase
